# Multicolored MIS-C, a single-centre cohort study

**DOI:** 10.1186/s12887-023-03997-0

**Published:** 2023-04-21

**Authors:** Petra Varga, András Balajthy, Erika Biró, Bernadett Bíró, Zsolt Reiger, Edit Szikszay, Gábor Mogyorósy, Rita Káposzta, Tamás Szabó

**Affiliations:** Department of Pediatrics, Faculty of Medicine, University of Debrecen, Nagyerdei krt 98, 4032 Debrecen, Hungary

**Keywords:** Multisystem inflammatory syndrome in children, MIS-C, COVID-19 in children, SARS-CoV-2

## Abstract

**Background:**

The aim of this study was to investigate the clinical and laboratory parameters that can predict the severity of Multisystem Inflammatory Syndrome in Children (MIS-C) at admission.

**Methods:**

We conducted a single-center, partly retrospective, partly prospective, observational cohort study between November 1, 2020 and December 31, 2021, which included patients aged from 1 month to 19 years, meeting the diagnostic criteria of MIS-C. We categorized the patients into three subgroups based on clinical and laboratory markers and assessed the predictive value of these factors in terms of ICU administration and cardiac abnormalities.

**Results:**

53 patients were classified in the following subgroups: Kawasaki-like disease (group 1) (47.2%, n = 25), shock with or without acute cardiac dysfunction (group 2) (32%, n = 17), fever and inflammation (group 3) (20.8%, n = 11). Subgroup analysis revealed that patients with shock and KD at initial presentation had significantly more severe manifestation of MIS-C requiring intensive care unit (ICU) treatment. Of the initial laboratory values, only CRP showed a significant difference between the 3 clinical groups, being lower in group 3. 52.6% of patients were admitted to the ICU. The median length of ICU stay was 3 days (range 3–20). ICU admission was more likely in patients with shortness of breath, renal failure (AKI) and patients with significantly increased concentrations of ferritin, D-dimer, INR and significantly milder increase concentration of fibrinogen. We found that fibrinogen and ferritin levels are independent risk factors for ICU admission. Cardiac abnormalities were found in 56.6% of total (30/53), with the following findings: decreased left ventricular function (32%), coronary abnormality (11.3%), pericardial effusion (17%), arrhythmia (32.1%) and mitral regurgitation (26.6%). Diarrhea and conjunctivitis at the initial presentation with significantly elevated CRP, Pro-BNP and blood pH concentrations were found to be a potential predisposing factor for decreased cardiac function while Pro-BNP and pH were independent risk factors for MIS-C. Regardless of the initial symptoms of MIS-C, the outcome was generally favorable.

**Conclusions:**

Clinical characteristics and baseline laboratory values ​​may help identify patients at increased risk for severe disease outcome, such as need for intensive care, presence of shock and decreased cardiac function.

**Trial registration:**

Participation consent was not reqired and ethical considerations were unnecessary, since we did not perform any extra interventions, only the necessary and usual therapeutic and diagnostic methods were used.

## Background

The global pandemic caused by severe acute respiratory syndrome coronavirus 2 (SARS-CoV-2), which leads to COVID-19, has rapidly spread worldwide, and although children have been relatively spared, a rare but severe hyperinflammatory condition known as multisystem inflammatory syndrome in children (MIS-C) or pediatric inflammatory multisystem syndrome temporally associated with SARS-CoV-2 (PIMS-TS) has been observed since April 2020 in Europe [1–3]. This condition typically occurs four weeks after a mostly asymptomatic or slightly symptomatic SARS-CoV-2 infection, and it is characterized by a variety of symptoms that overlap with Kawasaki disease (KD), toxic shock syndrome (TSS), sepsis, and macrophage activation syndrome (MAS) [3–5].

New, mostly overlapping case definitions helped identify patients with MIS-C [6–8]. For the proper diagnosis parallel inflammation in laboratory tests and multiorgan involvement have to be confirmed, including cardiac, gastrointestinal, haematological, mucocutaneous, neurological, respiratory, and renal systems. It is important to exclude other alternative diseases like TSS, KD or sepsis and prove a link to SARS-CoV-2 infection [6–8].

Series of cases and summary reports from around the world help determine the most common clinical features of MIS-C [9–12]. Gastrointestinal symptoms are common and prominent, occurring in 60–100% of children with MIS-C, which may even lead to laparoscopy due to suspicion of appendicitis [13, 14]. Most children with MIS-C have cardiovascular complications such as shock, decreased cardiac function, and coronary artery dilatation [15]. Approximately 60% of patients are admitted to intensive care unit (ICU) and the mortality rate can reach 2% [11].

The identification of prognostic factors that are associated with a poor outcome in multisystem inflammatory syndrome in children (MIS-C) is crucial for initiating early treatment decisions and the use of immunomodulatory therapy [16–19]. Despite recent studies that have proposed plausible prognostic factors for severe outcomes, high-level evidence is still lacking [20, 21].

The objective of this study is to provide a comprehensive description of the clinical, laboratory, and radiological characteristics, as well as the outcomes, of a larger cohort of children with MIS-C. The study aims to facilitate the early identification of critically ill patients with MIS-C, with the ultimate goal of improving their outcomes.

## Materials and methods

### Study design and patient selection

This was a single-centre (Department of Pediatrics, University of Debrecen), partly retrospective, partly prospective, observational cohort study conducted between November 1, 2020 and December 31, 2021 on patients from 1 months to 19 years who met the diagnostic criteria of MIS-C as defined by Centers for Disease Control and Preventation (CDC) [7]. The US CDC case definition was used to identify patients with MIS-C where all 4 criteria must be met: (I) age: <21 years), (II) Clinical presentation: fever > 38.0 °C for ≥ 24 h AND laboratory evidence of inflammation (any of the followings: elevated C-reactive protein (CRP), erythrocyte sedimentation rate (ESR), fibrinogen, procalcitonin (PCT), D-dimer, ferritin, lactic acid dehydrogenase (LDH), interleukin 6 (IL-6), neutrophils, reduced lymphocytes, low albumin) AND severe illness requiring hospitalization, with multisystem (2 or more than 2) organ involvement (cardiovascular, respiratory, renal, neurologic, hematologic, gastrointestinal, dermatologic) (III) no alternative plausible diagnoses; (IV) current or recent SARS-CoV-2 infection diagnosed by a positive reverse transcription polymerase chain reaction (RT-PCR) or positive antigen test or positive serological tests (IgM, IgG or IgA), or exposure to a suspected or confirmed COVID-19 case (epidemiologic link) within four weeks prior to the onset of symptoms.

In this study, we divided patients into three subgroups, with the aim of identifying clinical characteristics at the time of admission that could predict severe outcomes and searching for prognostic factors among patients admitted to the intensive care unit or with cardiac dysfunction. The three subgroups were as follows: Group 1, KD-like patients were diagnosed using the American Heart Association (AHA) definition with or without the presence of shock or myocardial dysfunction which included classic type (persistent fever ≥ 5 days together with at least 4 of the 5 principal clinical features), and incomplete type (persistent fever ≥ 5 days together with 2 or 3 of the 5 principal clinical features plus additional laboratory or echocardiographic findings) [22]; Group 2, patients with different types of shock (defined as hypotension, reliance on vasoactive agents to maintain normotension, or signs of inadequate tissue perfusion such as prolonged capillary refill time, oliguria, metabolic acidosis, or elevated serum lactate), with or without cardiac dysfunction (defined as ejection fraction < 55%), who did not fulfill KD criteria; and Group 3, patients with fever and inflammation, who did not meet either KD criteria or symptoms of shock.

### Data collection

Demographic, clinical, epidemiological, radiological, laboratory and outcome data were partly retrospectively, partly prospectively collected including: (1) demographic characteristics: age, sex; (2) medical history of comorbidity, obesity (body mass index (BMI): according to the WHO child growth standards, weight/age ratio); (3) main laboratory findings at presentation: platelet count, white blood cell count, lymphocyte count, CRP, PCT, IL-6, D-dimer, fibrinogen, cardiac troponin-T, Pro-B type natriuretic peptide (pro-BNP), ferritin, blood gas values (4) main clinical symptoms at presentation: fever, mucocutaneous involvement, nonsuppurative conjunctivitis, gastrointestinal symptoms, respiratory symptoms, cardiovascular symptoms, neurologic symptoms, renal symptoms; (5) electrocardiogram (ECG) abnormality at presentation and during treatment, echocardiography within 24 h of admission and subsequently during treatment; (6) testing for SARS-CoV-2: RT-PCR using oro/nasopharyngeal swabs or tracheal aspirates and/or antigen test using oro/nasopharyngeal swabs and/or serological test. COVID-19 serology testing was performed by two immunoassays: Cobas® anti-SARS-CoV-2 Ig tests were used to quantify total SARS-CoV-2 antibodies against N-protein and S-RBD protein, respectively (Roche Diagnostics, Mannheim, Germany). These tests consisted of electro-chemiluminescence indirect assay (ECLIA) and included recombinant N or S-RBD antigens, which bound serum antibodies in a double-antigen sandwich setup. Seropositivity was evaluated based on the manufacturer’s cut-off values of 1.0 (COI) and 0.8 U/mL, respectively. (7) clinical outcome: the length of time spent in the intensive care unit, days of oxygen requirement, inotropic support, mortality, short-term (6-month follow-up studies) consequences.

### Data processing and statistical analysis

Demographic, clinical, laboratory and outcome data were analyzed in all 3 groups both on the basis of clinical presentation (Kawasaki-like disease, different types of shock, fever and inflammation) and the need of ICU observation/treatment as well as the lack or presence of acute cardiac dysfunction. Continuous variables were described as medians and interquartile ranges (IQRs) or (mean and ER) and categorical variables as frequencies and percentages. Laboratory values ​​were also reported as the proportion of aberrant values. Numeric variables were compared using unpaired Student’s T-test or one-way analysis of variance (ANOVA). In case of categorical variables, we performed Chi square (or Fisher’s exact test). For the post hoc tests we applied Bonferroni correction method. We performed univariate regression analysis on all the potential risk factors. For multivariate logistic regression model we used the variables with a p value lower than 0.1 at univariate level. We calculated the crude odds ratios (OR) at univariate level, and adjusted OR values at multivariate level with 95% confidence interval (CI) to demonstrate the differences in regression analysis. We used SPSS V.25 program for statistical calculations. Statistical significance was accepted when two-sided p was < 0.05.

## Results

Nationwide, the number of children diagnosed with MIS-C has accumulated since October 2020 due to a significant increase in the number of acute cases observed in the second wave of the SARS-CoV-2 pandemic. Until 31 December 2021, 53 patients met the MIS-C definition (CDC, 2020) in our institute [7]. There was no death in our patient population. No complications were found on the short-term 6-month follow-up studies. We summarized demographic and clinical parameters of the total study population in Table 1.

**Table 1:** Demographic characteristics, clinical features, laboratory findings and clinical outcome of all MIS-C patients

	All patients (n = 53)	
Demographic and clinical characteristics at admission, n (%)		
Age in years, median (min.-max.)	7 (5 weeks − 17 years)	
Male sex	32 (60.4%)	
Comorbidities, n (%)	11 (20.8%)	
Fever, days at admission, median	5	
Gastrointestinal symptoms	38 (71.7%)	
Mucocutaneous symptoms	40 (75.5%)	
Cardiovascular symptoms	30 (56.6%)	
Respiratory symptoms	19 (35.8%)	
Neurological symptoms	5 (9.4%)	
Renal symptoms	9 (17%)	
**SARS-CoV-2 test results, n (%)**		
Positive RT-PCR	12 (22.6%)	
Positive serology	42/51 (82.4%)	
Laboratory confirmed SARS-CoV-2 infection	47 (88.7%)	
Epidemiological link to confirm SARS-CoV-2 infection	20 (37.7%)	
**Cardiogical disorders, n (%)**		
Myocardial dysfunction	17 (32%)	
Coronary artery abnormalities	6 (11.3%)	
Coronary artery aneurysm	0	
Mitral valve regurgitation	14 (26.6%)	
Pericardial effusion	9 (17%)	
Abnormal ECG	17 (32.1%)	
**Laboratory test at admission (normal range), median (IQR), % of aberrant values**		
Platelets (150–400 Giga/L)	248 (140–332)	43.40%
White blood cell count (4.5–11.5 Giga/L)	12 (9.23–17.07)	58.49%
Absolute lymphocyte count (0.9-4 Giga/L)	1.43 (0.95–2.84)	28.30%
C-reactive protein (< 2.2 mg/L)	171.91 (103.16-211.74)	98.11%
PCT (< 0.5 ug/L)	1.83(0.72–3.96)	75.47%
Interleukin-6 (< 7 ng/L)	186.8 (70.57-331.45)	92.45%
Cardiac TnT (< 10 ng/L)	19.83 (4.78–35.64)	67.92%
Pro-BNP (< 191.1 ng/L) < 191,1	2609.83 (935.34-9108.19)	79.25%
Ferritin (13–150 ug/L)	420 (230.5-626.9)	84.91%
INR	1.035 (0.96–1.11)	79.25%
D-dimer (< 0.5 mg FEU/L)	2.42 (1.48–4.25)	N/A
Albumin (35–52 g/L)	34 (30–36)	92.45%
Urea (1.4–6.8 mmol/L)	4.2 (3.6–6.4)	45.28%
Creatinine (26–88 umol/L)	42 (33–55)	20.75%
GPT (< 40 U/L)	20.5 (14-35.25)	7.55%
Bicarbonate (24 mmol/L)	24.8 (22.82–26.32)	18.87%
**Clinical outcome, n (%)**		
Days of pediatric intensive care unit (median)	3	
Oxygen therapy	8 (15.1%)	
Non-invasive respiratory support	3 (5.7%)	
Invasive mechanical ventilation	2 (3.8%)	
Vasoactive drugs	18 (34%)	
Death	0	

Association with SARS-CoV-2 infection was confirmed in 47 patients (88.7%): 12 with positive RT-PCR (22.6%), 42 with positive serology (42/51, 82.4%). In 7 out of the 53 patients (13.2%), both tests were positive. In 6 patients (11.3%) with negative laboratory tests for both RT-PCR and serology, only an epidemiological link was confirmed (Table 1).

Retrospectively, patients were divided into 3 groups based on the leading clinical symptoms at the first visit. The groups were as follows: Kawasaki-like disease 47.2% (group 1) (n = 25), shock with or without acute cardiac dysfunction 32% (group 2) (n = 17), fever and inflammatory group 20.8% (group 3) (n = 11) (Table 2).

**Table 2:** Demographic and clinical characterstics, laboratory test results and outcome data in the three clinical subgroups of MIS-C patients based on the leading clinical symptoms at the first visit

	Kawasaki-like group (1) n = 25 (47.2%) KD: 14, IKD: 11		Shock group (2) n = 17 (32%)		Fever, inflammatory group (3) n = 11 (20.8%)			p
Demographic and clinical characteristics at admission, n (%)								
Age in years, median (min.-max.)	6 (3–10)		9 (3–17)		6 (19 months − 15 years)			0.49
Male sex	11 (44%)		11 (64.7%)		10 (90.9%)			0.027
Comorbidities, n (%)	4 (16%)		3 (17.6%)		4 (36.4%)			0.97
Fever, days at admission, median	6		5		4			0.144
Gastrointestinal symptoms	19 (76%)		13 (76.5%)		6 (54.5%)			0.09
Mucocutaneous symptoms	25 (100%)		12 (70.6%)		2 (18.2%)			< 0.001
Cardiovascular symptoms	14 (56%)		13 76.5%)		3 (27.3%)			0.078
Respiratory symptoms	8 (32%)		6 (35.3%)		5 (45.5%)			0.333
Neurological symptoms	1 (4%)		2 (11.8%)		2 (18.2%)			0.547
Renal symptoms	1 (4%)		6 (35.3%)		4 (36.4%)			0.030
**SARS-CoV-2 test results, n (%)**								
Positive RT-PCR	4 (16%)		4 (23.5%)		4 (36.4%)			0.093
Positive serology	21/24 (87.5%)		12 (70.6%)		9 (81.8%)			0.198
Laboratory confirmed SARS-CoV-2 infection	24 (96%)		13 (76.5%)		10 (90.9%)			0.091
Epidemiological link to confirmSARS-CoV-2 infection	11 (44%)		6 (35.3%)		3 (27.3%)			0.386
**Cardiogical disorders, n (%)**								
Myocardial dysfunction	6 (24%)		11 (64.7%)		0			0.037
Coronary artery abnormalities	3 (12%)		1 (5.9%)		1 (9.1%)			0.80
Coronary artery aneurysm	0		0		0			N.A.
Mitral valve regurgitation	10 (25%)		4 (23.5%)		0			0.151
Pericardial effusion	4 (16%)		4 (23.5%)		1 (9.1%)			0.933
Abnormal ECG	7 (28%)		9 (52.9%)		0			0.182
**Laboratory test at admission (normal range), median (IQR), % of aberrant values**								
Platelets (150–400 Giga/L)	236 (128–330)	52.00%	216 (122–274)	47.06%	275 (250–347)	18.18%		0.683
WBC count (4.5–11.5 Giga/L)	12.84 (9.23–17.15)	68.00%	12 (9.71–14.43)	58.82%	11.31 (9.16–16.87)	36.36%		0.969
Absolute lymphocyte count (0.9-4 Giga/L)	1.395 (0.95–2.84)	36.00%	1.28 (0.9–1.75)	29.41%	2.32 (1.69–3.21)	9.09%		0.185
C-reactive protein (< 2.2 mg/L)	186.16 (133.21-243.13)	100.00%	174.12 (115,8-205)	100.00%	43 (28.73-139.14)	90.91%		0.003
PCT (< 0.5 ug/L)	1.91 (0.7–3.77)	80.00%	2.6 (0.87–8.09)	88.24%	0.9 (0.2–2.3)	45.45%		0.272
Interleukin-6 (< 7 ng/L)	211.4 (124.1-358.37)	100.00%	144.95 (51.52–299.5)	94.12%	100.21 (22.89-215.05)	72.73%		0.504
Cardiac TnT (< 10 ng/L)	23.21 (19.05–49.98)	60.00%	21.175 (14.13–59.79)	82.35%	29.3 (19.24–50.42)	63.64%		0.431
Pro-BNP (< 191.1 ng/L) < 191,1	1634.74 (829.8-7590.16)	80.00%	2909 (2400.94-1045.88)	88.24%	3016.61 (678.17-6425.63)	63.64%		0.803
Ferritin (13–150 ug/L)	396.2 (262.05-642.75)	80.00%	559.8 (385.9-703.6)	100.00%	188.3 (124.8-356.1)	45.45%		0.147
INR	1.03 (0.96–1.11)	N/A	1.06 (0.97–1.11)	N/A	1.04 (0.98–1.08)	N/A		0.248
D-dimer (< 0.5 mg FEU/L)	2.42 (1.53–3.84)	88.00%	3.58 (1.7–6.44)	100.00%	1.89 (0.7–3.62)	90.91%		0.258
Albumin (35–52 g/L)	34 (30–38)	44.00%	32.5 (28.75–35.25)	58.82%	36 (34–38)	27.27%		0.307
Urea (1.4–6.8 mmol/L)	4.55 (3.7–7.4)	24.00%	5.15 (3.77–8.02)	29.41%	3.8 (2.55–4.55)	0.00%		0.072
Creatinine (26–88 umol/L)	39.5 (33-52.75)	4.00%	48.5 (37-72.5)	17.65%	40 (28.5–52.5)	0.00%		0.144
GPT (< 40 U/L)	28 (19–60)	24.00%	23 (14-39.75)	23.53%	14 (12-18.5)	0.00%		0.335
Bicarbonate (24 mmol/L)	24.45 (22.97–25.5)	8.00%	25.75 (23.55–27.9)	17.65%	24.1 (22.17–25.92)	18.18%		0.476
**Clinical outcome, n (%)**								
Days of pediatric intensive care unit (median)	0		4		0		N/A	
Oxygen therapy	4 (16%)		3 (17.6%)		1 (9.1%)		0.97	
Non-invasive respiratory support	0		2 (11.8%)		1 (9.1%)			0.1106
Invasive mechanical ventilation	0		1 (5.9%)		0			0.16
Vasoactive drugs	6 (24%)		12 (70.6%)		0			0.049
Death	0		0		0			N/A

Patients in the KD group (n = 25, 47.2%) had either complete (n = 14, 56%) or incomplete KD (n = 11, 44%). 6 patients out of 25 required vasoactive drugs (24%), while 10 needed ICU treatment (40%). In patients with KD phenotype, presence of laboratory-confirmed SARS-CoV-2 infection was almost 100% (24/25) (Table 2). The leading symptom in 17 out of 53 patients (32%) was shock due to acute cardiac dysfunction (n = 11, 64.7%) or toxic shock-like syndrome (n = 6, 35.3%). In the group with shock, the median age tends to be higher (9 years), but not significantly compared to other groups. Within the shock group, the most commonly affected organ systems were the cardiovascular (100%) and gastrointestinal (76.5%). Patients had markedly elevated PCT, ferritin, D-dimer and decreased platelet count and albumin level, but these were not statistically significant compared to other groups. Beyond the need of fluid resuscitation (10 ml/kg iv. bolus over 5–10 min) in some cases, 70.6% of the patients required vasoactive drugs (Table 2). In 11 patients (20.8%) predominance of fever and inflammation (did not meet neither KD criteria nor symptoms of shock) was observed in association with other organ (most commonly renal, neurological) involvement. In contrast to the two other groups, COVID-19 RT-PCR positivity (36.4%) and the presence of respiratory symptoms seemed more common in this group (Table 2). Subgroup analysis revealed that patients with shock and KD at initial presentation had significantly more severe manifestation of MIS-C often requiring ICU treatment. However, no significant difference in length of ICU admission was found between patients of either group treated in the ICU. Of the initial laboratory values, only CRP showed a significant difference between the 3 clinical groups, as it was lower in group 3 (group (I) vs. group (II) p = 1; group (I) vs. group III. p = 0.005; group (II) vs. group III.p = 0.021).

We found significant differences in the clinical and laboratory parameters of the potential ICU and non-ICU patients (Table 3).

**Table 3:** Demographic, clinical characterstics and laboratory test results at admission and outcome data of the ICU and non-ICU patients

	Patients never admitted to ICU n = 23 (43.4%)		Patients admitted to ICU n = 30 (52.6%)		p
Demographic and clinical characteristics at admission, n (%)					
Age in years, median (min.-max.)	6.5 (19 months-17 years)		8 (5 weeks-17 years)		0.586
Male sex	12 (52.2%)		20 (66.7%)		0.337
Comorbidities, n (%)	6 (26.1%)		5 (16.7%)		0.501
Fever, days at admission, median	5		5		0.8
Gastrointestinal symptoms	14 (60.9%)		24 (80%)		0.2
Mucocutaneous symptoms	17 (73.9%)		23 (76.7%)		0.922
Cardiovascular symptoms	13 (56.5%)		17 (56.7)		0.528
Respiratory symptoms	4 (17.4%)		15 (50%)		0.085
Neurological symptoms	1 (4.3%)		4 (13.3%)		0.999
Renal symptoms	1 (4.3%)		8 (26.7%)		0.005
**SARS-CoV-2 test results, n (%)**					
Positive RT-PCR	4 (17.4%)		8 (26.7%)		0.613
Positive serology	18/22 (81.8%)		24/29 (82.8%)		0.723
Laboratory confirmed SARS-CoV-2 infection	19 (82.6%)		28 (93.3%)		0.385
Epidemiological link to confirm SARS-CoV-2 infection	9 (39.1%)		11 (36.7%)		0.579
**Cardiogical disorders, n (%)**					
Myocardial dysfunction	13 (56.5%)		17 (56.7%)		0.788
Coronary artery abnormalities	2 (8.7%)		4 (13.3%)		0.687
Coronary artery aneurysm	0		0		N.A.
Mitral valve regurgitation	7 (30.4%)		7 (23.3%)		0,79
Pericardial effusion	3		6		0.715
Abnormal ECG	3 (13%)		6 (20%)		0.715
**Laboratory test at admission (normal range), median (IQR), % of aberrant values**					
Platelets (150–400 Giga/L)	276 (224–365)	34.78%	235 (121.25-273.75)	48.39%	0.284
WBC count (4.5–11.5 Giga/L)	15.3 (9.46–17.51)	0.00%	11.28 (7.97–14.46)	54.84%	0.098
Absolute lymphocyte count (0.9-4 Giga/L)	1.77 (1.23–3.03)	21.74%	1.2 (0.9–1.97)	32.26%	0.993
C-reactive protein (< 2.2 mg/L)	167.14 (103.32-229.71)	91.30%	170.6 (104.04-205.63)	100.00%	0.813
PCT (< 0.5 ug/L)	1.445 (0.4–3.08)	56.52%	2.6 (0.87–6.27)	87.10%	0.2
Interleukin-6 (< 7 ng/L)	199.15 (109.17-315.25)	82.61%	202.9 (52.78–332.9)	96.77%	0.736
Cardiac TnT (< 10 ng/L)	23.79 (17.14–37.85)	56.52%	20.62 (10.17–54.79)	74.19%	0.666
Pro-BNP (< 191.1 ng/L) < 191.,1	2396.71 (224.53-7320.38)	65.22%	2759.415 (1565.81-9188.5)	87.10%	0.718
Fibrinogen (1.5-4 g/L)	7.97 (6.7–8.65)	78.26%	6.81 (4.53–8.07)	87.10%	0.031
Ferritin (13–150 ug/L)	355.3 (188.3-482.1)	69.57%	546.8 (378.45-762.05)	83.87%	0.034
INR	1.01 (0.96–1.04)	N/A	1.09 (0.98–1.14)	N/A	0.047
D-dimer (< 0.5 mg FEU/L)	1.615 (1.28–2.29)	82.61%	3.82 (2.28–6.52)	96.77%	0.032
Albumin (35–52 g/L)	36 (31.75-40)	39.13%	32.5 (29-35.75)	48.39%	0.084
Urea (1.4–6.8 mmol/L)	3.85 (3.12–6.1)	13.04%	5 (3.8–6.9)	25.81%	0.127
Creatinine (26–88 umol/L)	38.5 (33-46.75)	0.00%	48 (31–59)	12.90%	0.181
GPT (< 40 U/L)	19.5 (13.75-28)	13.04%	23 (14-39.75)	22.58%	0.413
Bicarbonate (24 mmol/L)	23.35 (22.45–25.5)	4.35%	25.05 (23.62–27.1)	19.35%	0.807
**Clinical outcome, n (%)**					
Days of pediatric intensive care unit (median)	0		5		N/A.
Oxygen therapy	1 (4.3%)		7 (23.3%)		0.118
Respiratory support	0		5 (16.67%)		0.001
Vasoactive drugs	0		18 (60%)		N/A
Death	0		0		N/A

**Fig. 1:**
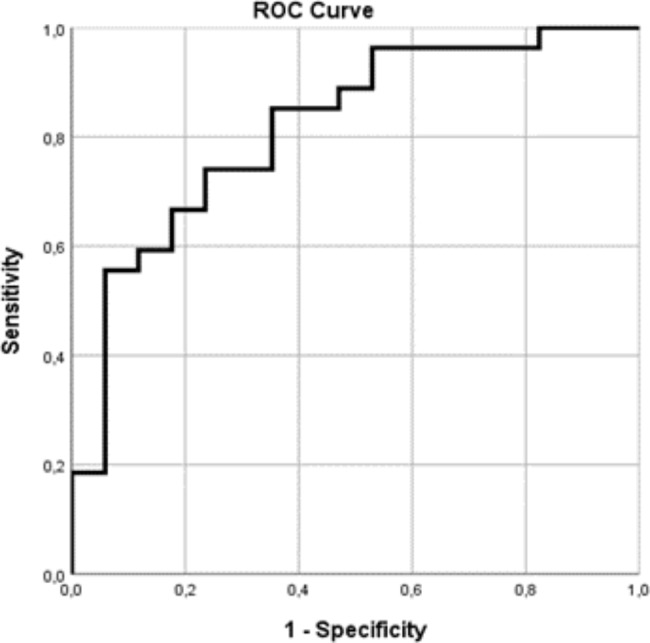
To demonstrate the predictive power of our regression model, we created a ROC curve using the predictive probabilities from the multivariate logistic regression model (Table 4). The area under the ROC curve was 0.817

A forward stepwise logistic regression analysis was performed to find clinical variables, which predict the necessity of ICU hospitalization as an indirect mode to quantify the severity of MIS-C. We used the variables in which p value was lower than 0.1 at univariate level. We found that fibrinogen and ferritin levels are independent risk factors for ICU admission. Our model explained 48.8% (Nagelkerke R2) of the variance and correctly classified 64.3% of cases. To demonstrate the predictive power of our model we created an ROC curve using the predictive probabilities from logistic regression model. The area under the ROC was 0.817 indicating a good prediction power (Table 4; Fig. 1).

**Table 4:** Independent risk factors for ICU admission based on multivariate regression analysis

Independent risk factors for ICU admission				
	p	Adjusted OR	95% C.I.	
fibrinogen	0.04	0.563	0.325	0.974
ferritin	0.048	1.003	1	1.006

Cardiac abnormalities were found in 56.6% of the total population. Myocardial dysfunction (left ventricular systolic function with an ejection fraction below 55%, which was calculated based on M-mode measurements) was detected in 17 patients (32%). Coronary artery dilatation could be detected (Z-score > 2.5) in 4 out of 53 patients (11.3%), all of which were reversible. Coronary artery aneurysm did not develop in our patients. Mitral valve regurgitation occurred in a total of 14 cases (26.6%), which recovered during follow up. Pericardial effusion was detected in 9 patients (17%), one of whom was at risk of tamponade. Arrhythmias such as sinus node depression and conduction disturbances were observed often as first symptom during the course, with a total of 17 patients (32.1%) experiencing ECG abnormalities (Table 1). Separate examination of potential predisposing factors for decreased cardiac function revealed that diarrhea (15.2% and 40%, respectively) and conjunctivitis (45.5% and 75%, respectively) occurred more commonly in these patients in parallel with elevated CRP, Pro-BNP concentrations, and pH (Table 5).

**Table 5:** Demographic, clinical characteristics and laboratory test results at admission and outcome data of the patients with or without decreased cardiac function

	Cardiac dysfunction n = 20 (37.7%)	Without cardiac dysfunction n = 33 (62.3%)	p
Demographic and clinical characteristics at admission, n (%)			
Age in years, median (min.-max.)	8	6	0.003
Male sex	14 (70%)	18 (54.5%)	0.265
Comorbidities, n (%)	1 (5%)	10 (30.3%)	0.037
Fever, days at admission, median	5.5	5	0.723
Gastrointestinal symptoms	14 (70%)	24 (72.8%)	0.831
Diarrhea	8 (40%)	5 (15.2%)	0.042
Mucocutaneous symptoms	17 (85%)	23 (69.7%)	0.209
Conjunctivitis	15 (75%)	15 (45.5)	0.035
Cardiovascular symptoms	20 (100%)	10 (30.3%)	< 0.001
Respiratory symptoms	5 (25%)	14 (42.4%)	0.765
Neurological symptoms	0	5 (15.2%)	0.105
Renal symptoms	3 (15%)	6 (18.2%)	0.765
**SARS-CoV-2 test results, n (%)**			
Positive RT-PCR	4 (20%)	8 (24.2%)	0.721
Positive serology	18 (90%)	24/31 (77.4%)	0.250
Laboratory confirmed SARS-CoV-2 infection	18 (90%)	29 (87.9%)	1.00
Epidemiological link to confirm SARS-CoV-2 infection	8 (40%)	12 (36.4%)	0.95
**Cardiogical disorders, n (%)**			
Myocardial dysfunction	19 (95%)	0	N.A.
Coronary artery abnormalities	2 (10)	4 (12.1%)	N.A.
Coronary artery aneurysm	0	0	N.A.
Mitral valve regurgitation	9 (45%)	5 (15.2%)	N.A.
Pericardial effusion	3 (15%)	6 (18.2%)	N.A.
Abnormal ECG	13 (65%)	4 (12.1%)	N.A.
**Laboratory test at admission (normal range), median (IQR)**			
Platelets (150–400 Giga/L)	237.5 (135.5-329.25)	252 (208–343)	0.335
WBC count (4.5–11.5 Giga/L)	13.565 (9.65–17.06)	11.45 (8.87–17.07)	0.335
Absolute lymphocyte count (0.9-4 Giga/L)	1.385 (1-2.26)	1.61 (0.95–2.84)	0.125
C-reactive protein (< 2.2 mg/L)	188.53 (64.19-232.99)	133.21 (88.76-205.85)	0.176
PCT (< 0.5 ug/L)	1.69 (0.86–5.53)	1.83 (0.51–3.625)	0.349
Interleukin-6 (< 7 ng/L)	174.1 (102.95-286.45)	189.15 (68.17–489.3)	0.005
Cardiac TnT (< 10 ng/L)	26.2 (19.68–88.46)	21.47 (12.82–36.5)	0.002
Pro-BNP (< 191.1 ng/L) < 191.1	9268.875 (2406.22-17237.48))	1634.74 (701.21-5085.19)	0.006
Ferritin (13–150 ug/L)	500.5 (379.05–692.3)	355.7 (169.47–598.4)	0.651
INR	1 (0.955–1.11)	1.04 (0.98–1.11)	0.309
D-dimer (< 0.5 mg FEU/L)	2.575 (1.53–3.98)	2.16 (1.4–4.88)	0.180
Albumin (35–52 g/L)	32 (29-35.75)	35 (31.75–38.25)	0.517
Urea (1.4–6.8 mmol/L)	5.15 (3.7–6.73)	4.2 (3.45-6)	0.844
Creatinine (26–88 umol/L)	42 (34.5–49)	44 (32-57.5)	0.185
GPT (< 40 U/L)	22 (14.25–29.5)	19.5 (12.25–36.75)	0.149
Bicarbonate (24 mmol/L)	25.75 (23.57–27.45)	24.25 (22.6–25.5)	0.804
pH (7.4)	7.465 (7.45–7.49)	7.42 (7.39–7.45)	0.008
**Clinical outcome, n (%)**			
Days of pediatric intensive care unit (median)	3 (15%)	2 (6.1%)	0.428
Respiratory support	1 (5%)	4 (12.1)	0.721
Vasoactive drugs	11 (55%)	7 (21.2%)	0.027
Death	0	0	N/A

**Fig. 2:**
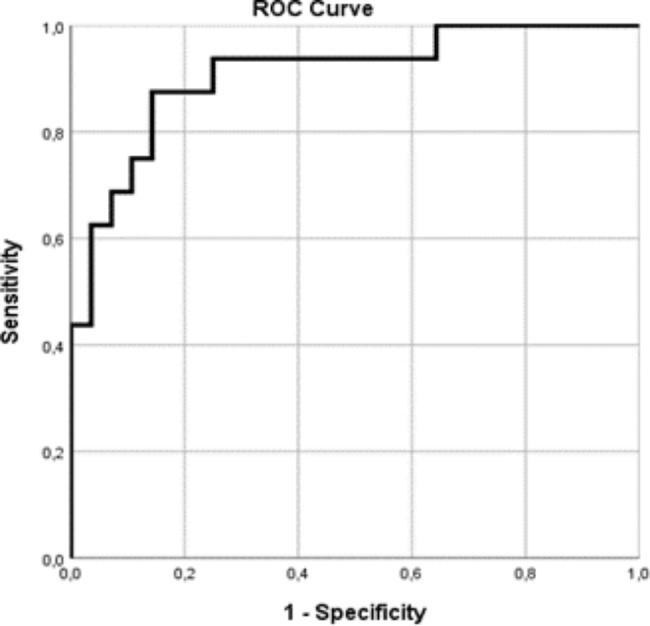
To demonstrate the predictive power of our regression model, we created a ROC curve using the predictive probabilities from the multivariate logistic regression model (Table 6). The area under the ROC curve was 0.953, which indicates good prediction power

To determine which factors are predictive for cardiac abnormalities in MIS-C we created a multivariate regression model using variables where p value was lower than 0.1 at univariate level. Our model showed that Pro-BNP and pH are independent risk factors for MIS-C. The model explained 62.9% (Nagelkerke R2) of the variance and correctly classified 84.1% of cases. To demonstrate the predictive power of our model we created a ROC curve using the predictive probabilities from the multivariate forward stepwise logistic regression model. The area under the ROC was 0.908 indicating a good prediction power (Fig. 2; Table 6).

**Table 6:** Independent risk factors for cardiac dysfunction based on multivariate regression analysis

Independent risk factors for cardiac dysfunction				
	p	Adjusted OR	95% C.I.	
pH	0.035	1.5*10^6^	12.642	1.78*10^29^
pro BNP	0.041	1.000	1.000	1.000

However, a cut-off value for Pro-BNP indicative of cardiac involvement could not be determined due to the small number of cases.

## Discussion

We present one of the first series of MIS-C cases from Hungary, with 52.6% of patients requiring admission to the ICU, which is consistent with other reports [11]. The number of reported MIS-C cases increased significantly during the second wave of the COVID-19 pandemic in Hungary, as seen in Western European countries. The rise in MIS-C cases typically occurred three to four weeks after the peak of COVID-19 cases within communities, as observed in most studies [2, 4, 5, 9].

Despite the severe course of the disease requiring intensive care in many children, the prognosis for MIS-C is generally favorable, with most children achieving complete clinical recovery. None of our patients died, which may be attributed to early diagnosis, adequate treatment, and timely preparation for MIS-C cases based on alerts from European and American notifications [4, 10, 11, 23, 24]. Both the median age of our patient cohort (7 years) and the sex distribution (60.4% male) are similar to that of other reports. Although obesity was the most common associated disease in most reports, it was not observed in our patients, in contrast to the more severe disease course seen in acute SARS-CoV-2 infection [4, 5, 10]. MIS-C related symptoms occurred with a similar frequency in our cohort as it was reported in relevant clinical reports presenting the clinical spectrum of the disease [12].

Given the wide range of clinical manifestation and outcome, the identification of factors associated with more severe outcome in patients with MIS-C may help assess prognosis and make early treatment decisions [4, 11, 20, 21]. We demonstrated that higher D-dimer, ferritin, INR and lower fibrinogen at admission indicate severe course, while higher CRP and Pro-BNP predispose to cardiac involvement in MIS-C. To identify MIS-C patients requiring ICU treatment, several studies and publications are seeking for prognostic factors at admission [20, 21, 25]. A meta-analysis of 787 MIS-C patients revealed that higher levels of leukocyte, absolute neutrophil count, CRP, D-dimer, and ferritin was detected in severe MIS-C patients compared with non-severe MIS-C patients [25]. In another retrospective surveillance study 1080 patients with MIS-C were enrolled. Intensive care was more likely to occur in patients with dyspnoea, abdominal pain, and elevated CRP, troponin, ferritin, D-dimer, Pro-BNP, or IL-6, or decreased platelet or lymphocyte counts [20]. A meta-analysis of 1613 patients showed higher levels of Pro-BNP indicative of cardiac involvement compared to patients with severe MIS-C than in patients with non-severe MIS-C [21, 26].

According to previous literature data, nearly 50% of patients (typically younger patients) belonged to the KD-like group. However, in contrast to the classic (typical) appearance of KD, KD-like MIS-C patients usually had more severe condition (40% required intensive care, 24% received shock management compared to similar entities of the “classic” form of KD, where shock is less common, occurs only in 5% of patients). In accordance with literature data, MIS-C patients tend to be older compared with classical KD patients [22]. Similarly to published case series, symptoms of gastrointestinal involvement were quite common in our cohort (76%), with typically elevated inflammatory parameters and lower platelet counts (Table 1) [27–30].Coronary heart disease was rare among patients with cardiac symptoms which observation is consistent with data of previous reports [2, 4, 5, 9, 31, 32].

Although, nearly 100% of patients in the shock group required therapy at ICU and 70.6% required vasoactive drugs, no statistically significant differences were found in this group in the initial laboratory studies (except for CRP). The above can be explained not only by the small number of cases but also by the large number of severe patients in the KD group [11].

In 11 patients (group 3) we observed fever and severe inflammation along with other organ involvement, typically renal or neurological involvement. Comparing to the two other groups the presence of respiratory symptoms was far more common in this particular group of patients (difference is not significant). The overlap with acute COVID-19 (36.4% RT-PCR positivity) may be a plausible explanation for that (Table 2) [11].

Subgroup analysis based on clinical presentation showed that baseline laboratory values (excluding CRP, which was significantly lower in group 3), did not differ significantly between the 3 groups. Prognostic factors for either severity or course alone cannot be determined from the initial clinical presentation.

There are several limitations to our study. This study was conducted in a single center, a similar multicenter study with greater power may help validate our results and remove the bias of the study.

## Conclusion

Children with MIS-C can present with a variety of clinical manifestations. Our study identified several signs and symptoms, including shortness of breath and renal symptoms, along with abnormal laboratory markers such as elevated ferritin, D-dimer, and INR, and decreased fibrinogen at admission that indicate a severe course and the need for intensive care in our patient population. Higher levels of CRP and Pro-BNP predispose to cardiac involvement in MIS-C. The prognosis of MIS-C is still uncertain, given the novelty of this clinical entity and the lack of long-term follow-up studies [33, 34]. Our results emphasize the importance of rapid diagnostic and therapeutic interventions to prevent long-term complications in patients with suspected MIS-C. Both clinical and laboratory prognostic factors can aid in identifying high-risk patients with a more severe disease course.

## Data Availability

Dataset analyzed during the study represent patient’s data available in their medical documentation and the electronic patients’ database (MedSolution, UDMed) of the University of Debrecen for authorized personnel. Petra Varga the first author can be contacted for additional data request (varga.petra@med.unideb.hu).

## Abbreviations

MIS-C: Multisystem Inflammatory Syndrome in Children
ICU: Intensive care unit
COVID-19: Coronavirus disease 2019
KD: Kawasaki disease
CRP: C-reactive protein
AKI: Acute kidney injury
INR: International Normalized Ratio
Pro-BNP: Pro-B-type natriuretic peptide
SARS-CoV-2: Severe Acute Respiratory Syndrome Coronavirus 2
PIMS-TS: Pediatric Inflammatory Multisystem Syndrome Temporally associated with SARS-CoV-2
TSS: Toxic shock syndrome
MAS: Macrophage activation syndrome
CDC: Centers for Disease Control and Prevention
ESR: Erythrocyte sedimentation rate
PCT: Procalcitonin
LDH: Lactic acid dehydrogenase
IL-6: Interleukin 6
RT-PCR: Reverse transcription polymerase chain reaction
BMI: Body mass index
WHO: World Health Organization
ECG: Electrocardiogram
IQRs: Interquartile ranges
OR: Odds ratio
CI: Confidence interval

## References

[1] Castagnoli R, Votto M, Licari A (2020). Severe acute respiratory syndrome coronavirus 2 (SARS-CoV-2) infection in children and adolescents: a systematic review. JAMA Pediatr.

[2] Verdoni L, Mazza A, Gervasoni A, Gervasoni A (2020). An outbreak of severe Kawasaki-like disease at the italian epicentre of the SARS-CoV-2 epidemic: an observational cohort study. Lancet.

[3] Riphagen S, Gomez X, Gonzalez-Martinez C (2020). Hyperinflammatory shock in children during COVID-19 pandemic. Lancet.

[4] Whittaker E, Bamford A, Kenny J (2020). Clinical characteristics of 58 children with a Pediatric Inflammatory Multisystem Syndrome temporally Associated with SARS-CoV-2. JAMA.

[5] Feldstein LR, Rose EB, Horwitz SM (2020). Multisystem inflammatory syndrome in U.S. children and adolescents. N Engl J Med.

[6] Royal College of Paediatrics and Child Health Guidance. : Paediatric multisystem inflammatory syndrome temporally associated with COVID-19 [Internet]. RCPCH. Available from: https://www.rcpch.ac.uk/resources/guidance-paediatric-multisystem-inflammatory-syndrome-temporally-associated-covid-19-pims. Accessed 20 Jul 2020.

[7] Centers for Disease Control and Prevention. Multisystem inflammatory syndrome in children (MIS-C) associated with coronavirus disease 2019 (COVID-19) [Internet]. 2020. Available from: https://emergency.cdc.gov/han/2020/han00432.asp. Accessed 20 Jul 2020.

[8] World Health Organization. Multisystem inflammatory syndrome in children and adolescents temporally related to COVID-19 [Internet]. [cited 2020 Jul 29]. Available from: https://www.who.int/news-room/commentaries/detail/multisystem-inflammatory-syndrome-in-children-and-adolescents-with-covid-19

[9] Dufort EM, Koumans EH, Chow EJ (2020). Multisystem inflammatory syndrome in children in New York State. N Engl J Med.

[10] Levi Hosta, Paemel RV, Haerynck F (2021). Multisystem inflammatory syndrome in children realated to Covid-19: a systematic review. Eur J Pediatr.

[11] Godfred-Cato S, Bryant B, Leung J (2020). COVID-19-Assiciated Multisystem Inflammatory Syndrome in Children – United States, March-July 2020. MMWR Morb Mortal Wkly Rep.

[12] Ahmed M, Advani S, Moreira A (2020). Multisystem inflammatory syndrome in children: a systematic review. EClinicalMedicine.

[13] Nakra NA, Blumberg DA, Herrera-Guerra A (2020). Multi-system inflammatory syndrome in children (MIS-C) following SARS-CoV-2 infection: review of clinical presentation, hypothetical pathogenesis, and proposed management. Children.

[14] Gerall CD, Duron VP, Griggs CL (2021). Multisystem inflammatory syndrome in Children Mimicking Surgical Pathologies what surgeons need to know about MIS-C. Ann Surg.

[15] Abrams JY, Godfred-Cato SE, Oster ME (2020). Multisystem inflammatory syndrome in children associated with severe acute respiratory syndrome coronavirus 2: a systematic review. J Pediatr.

[16] Henderson LA, Canna SW, Friedman KG (2021). American College of Rheumatology Clinical Guidance for Multisystem Inflammatory Syndrome in Children Associated with SARS-CoV-2 and Hyperinflammation in Pediatric COVID-19: version 2. Arthritis Rheumatol.

[17] American Academy of Pediatrics clinical guidance. : Multisystem Inflammatory Syndrome in Children (MIS-C), available from: https://services.aap.org/en/pages/2019-novel-coronavirus-covid-19-infections/clinical-guidance/multisystem-inflammatory-syndrome-in-children-mis-c-interim-guidance. Accessed on November 23, 2020.

[18] Harwood R, Allin B, Jones CE (2021). A national consensus management pathway for paediatric inflammatory multisystem syndrome temporally associated with COVID-19 (PIMS-TS): results of a national Delphi process. Lancet Child Adolesc Health.

[19] Henderson La, Canna SW, Friedman KG (2022). American College of Rheumatology: Clinical Guidance for Pediatric Patients with Multisystem Inflammatory Syndrome in Children (MIS-C) Associated with SARS-CoV-2 and Hyperinflammation in COVID-19: Version 3. Arthritis Rheumatol.

[20] Abrams JY, Matthew E, Oster MO, Shana E, Godfred-Cato SE (2021). Factors linked to severe outcomes in multisystem inflammatory syndrome in children (MIS-C) in the USA: a retrospective surveillance study. Lancet Child Adolesc Health.

[21] Zhao Y, Patel J, Huang Y (2021). Cardiac markers of multisystem inflammatory syndrome in children (MIS-C) in COVID-19 patients: a meta-analysis. Am J Emerg Med.

[22] McCrindle BW, Rowley AH, Newburger JW (2017). Diagnosis, treatment, and long-term management of Kawasaki Disease: A Scientific Statement for Health Professionals from the American Heart Association. Circulation.

[23] Jonat B, Gorelik M, Boneparth A (2021). Multisystem inflammatory syndrome in Children Associated with Coronavirus Disease 2019 in a children’s hospital in New York City: patient characteristics and an institutional protocol for evaluation, management, and Follow-Up. Pediatr Crit Care Med.

[24] Ouldali N, Toubiana J, Antona D (2021). Association of intravenous immunoglobulins plus methylprednisolone vs immunoglobulins alone with course of Fever in Multisystem Inflammatory Syndrome in Children. JAMA.

[25] Zhao Y, Yin L, Patel J, et al. The inflammatory markers of multisystem inflammatory syndrome in children (MIS-C) and adolescents associatedvwith COVID-19: A meta-analysis. J Med Virol. 2021; 93:4358–4369. 10.1002/jmv.26951 PMC8250955 33739452

[26] Wu J-R, Chen I-C, Dai Z-K, et al. Early elevated B-Type natriuretic peptide levels are Associated with Cardiac Dysfunction and Poor Clinical Outcome in Pediatric Septic Patients. Acta Cardiol Sin. 2015;31:485–93. 10.6515/ACS20141201E PMC4804972 27122912

[27] Consiglio CR, Cotugno N, Sardh F, Pou C (2020). The Immunology of Multisystem Inflammatory Syndrome in Children with COVID-19. Cell.

[28] Rowley AH (2020). Understanding SARS-CoV-2-related multisystem inflammatory syndrome in children. Nat Rev Immunol.

[29] Rodríguez-Rubio M, Menéndez-Suso JJ, Cámara-Hijón C et al. Cytokine Profile in Children with Severe Multisystem Inflammatory Syndrome Related to the Coronavirus Disease 2019.Online J Pediatr Intensive Care. February24, 2021. 10.1055/s-0041-1724101 PMC9345675 35928043

[30] Roasted CA, Chahroudi A, Mantus G (2020). Quantitative SARS-CoV-2 Serology in Children with Multisystem Inflammatory Syndrome (MIS-C). Pediatrics.

[31] Cheung EW, Zachariah P, Gorelik M (2020). Multisystem inflammatory syndrome related to COVID-19 in previously healthy children and adolescents in New York City. JAMA.

[32] Niaz T, Hope K, Fremed M et al. Role of a Pediatric Cardiologist in the COVID 19 Pandemic.Pediatr Cardiol. 2020; Oct4:1–17. 10.1007/s00246-020-02476-y PMC7533115 33015722

[33] Alsaied T, Tremoulet AH, Burns JC, Saidi A, Dionne A, Lang SM, Newburger JW, de Ferranti S, Friedman KG (2021). Review of Cardiac involvement in Multisystem Inflammatory Syndrome in Children. Circulation.

[34] Kaushik A, Gupta S, Sood M (2020). A systematic review of Multisystem Inflammatory Syndrome in Children Associated with SARS-CoV-2 infection. Pediatr Infect Dis J.

